# Eating disorder symptoms among transgender and gender diverse youth

**DOI:** 10.1177/13591045231184917

**Published:** 2023-06-21

**Authors:** Rachel Kramer, Claire M Aarnio-Peterson, Lee Ann Conard, Katrina R Lenz, Abigail Matthews

**Affiliations:** 1 Division of Behavioral Medicine and Clinical Psychology, 2518 Cincinnati Children’s Hospital Medical Center, Cincinnati, OH, USA; 2 Department of Psychiatry and Behavioral Sciences, 8785 University of California, San Francisco, San Francisco, CA, USA; 3 College of Medicine, Department of Pediatrics, 2514 University of Cincinnati, Cincinnati, OH, USA; 4 Division of Adolescent Medicine, 2514 Cincinnati Children’s Hospital Medical Center, Cincinnati, OH, USA; 5 Department of Child and Adolescent Psychiatry & Behavioral Sciences, 6567 Children’s Hospital of Philadelphia, Philadelphia, PA, USA

**Keywords:** Eating disorders, transgender and gender diverse, hormone replacement therapy, youth

## Abstract

Compared to cisgender peers, transgender and gender diverse (TGD) youth and adults report elevated eating disorder (ED) symptoms likely related to gender dysphoria and attempts to modify their bodies accordingly. Less is known about the impact on gender-affirming care and ED symptoms. This study aimed to expand on extant research and describe ED symptoms in TGD youth seeking gender-affirming care while exploring potential associations between gender-affirming hormone use and ED symptoms. A total of 251 TGD youth completed the Eating Disorders Examination-Questionnaire (EDE-Q) as part of routine clinical care. ANCOVAs and negative binomial regressions examined differences in ED symptoms among transgender females (identifying as female but assigned male at birth) and transgender males (identifying as male but assigned female at birth). ED severity was not significantly different among transgender females versus transgender males, (*p* = .09), or associated with gender-affirming hormone use (*p* = .07). Transgender females receiving gender-affirming hormones reported a greater proportion of objective binge eating episodes compared to those who were not (*p* = .03). Over a quarter of TGD youth reported engagement in ED behaviors suggesting assessment and intervention related to ED behaviors among TGD youth is imperative since adolescence is a particularly vulnerable period for adolescents and engagement in ED behaviors could lead to full ED development and medical risk.

## Introduction

Eating disorders (EDs) such as anorexia nervosa, atypical anorexia nervosa, and bulimia nervosa have devastating consequences, including medical complications (Rome & Ammerman, 2003), substantial treatment costs (Agras, 2001), and one of the highest mortality rates of any psychiatric disorder second to opiate use (Hudgins et al., 2019). Emerging research illustrates EDs impact diverse populations (Schaumberg et al., 2017) including transgender and gender diverse (TGD) individuals (Calzo et al., 2017; Jones et al., 2018).

Compared to cisgender adults, research suggests adults identifying as transgender (defined as having a gender identity incongruent with their sex assigned at birth) (Turban & Ehrensaft, 2018) self-identify as having an ED and endorse greater ED symptomatology, including body dissatisfaction, laxative and diet pill use, steroid abuse, self-induced vomiting, binge eating episodes, and fasting behaviors (Cusack et al., 2022; Diemer et al., 2015 Mirabella et al., 2020; Nagata et al., 2020; Vocks et al., 2009; Watson et al., 2017; Witcomb et al., 2015). In TGD adolescents and young adults (henceforth referred to as “TGD youth”; Assembly, U.G., 1989), studies illustrate 3.9%–5.6% seeking care at specialty transgender clinics present with full threshold EDs (Feder et al., 2017; Ferrucci et al., 2022). In other studies, 10% of treatment-seeking TGD youth reported restrictive eating, binge eating, and/or purging behaviors (Feder et al., 2017), 15% reported cliniically elevated ED symptoms, and over 50% had engaged in behaviors to manipulate weight and shape (Avila et al., 2019). Further, 25% of TGD youth seeking gender-affirming hormone use endorsed one or more binge eating episode in the last 28-days (Peterson et al., 2020).

Identifying as TGD, is not, in and of itself, a risk factor for psychological distress (McClain & Peebles, 2016). While TGD youth experience gender incongruence, which refers to having a gender identity that does not match one’s gender assigned at birth, not all TGD individuals experience gender dysphoria, defined as psychological distress in relation to a desire to be a different gender than assigned at birth (Turban & Ehrensaft, 2018). However, factors uniquely associated with identifying as TGD may present an additive risk for ED development (Brewster et al., 2019; Jones et al., 2018; McClain & Peebles, 2016; Stice, 2002; Jones et al., 2018; McClain & Peebles, 2016; Uniacke et al., 2021; van de Grift et al., 2016; Watson et al., 2017).

Adolescence is associated with the greatest prevalence of EDs (Ward et al., 2019) and this developmental period can be particularly difficult for TGD youth, especially those with gender dysphoria. The onset of puberty may prompt and exacerbate body development that is incongruent with one’s identified gender and foster body dissatisfaction, an established risk factor for EDs (Hembree et al., 2017; Jones et al., 2018; McClain & Peebles, 2016; van de Grift et al., 2016). Similarly, thin-and muscle-ideal internalization, also ED risk factors (Stice, 2002), may be prominent in TGD youth seeking more feminine or masculine-appearing bodies. For instance, Brewster and colleagues (2019) found that among transgender females (i.e., male sex assigned at birth and female gender identity), thin-ideal internalization predicted body dissatisfaction which predicted ED symptoms. Lastly, the experience of stigma and minority stress could also impact risk of ED development (Brewster et al., 2019; Cusack et al., 2021) through increased stress and vulnerability to psychological concerns placing youth at greater risk for ED development.

### Gender-affirming care and ED symptoms

Gender-affirming treatment is deemed the gold standard of care for TGD individuals (Coleman et al., 2022) and includes social (e.g., dressing as identified gender, name change), psychological (e.g., psychotherapy), and medical (e.g., gender-affirming surgeries, hormone replacement) interventions. Gender-affirming treatment is associated with the amelioration of gender dysphoria and general distress (Coleman et al., 2022; White Hughto & Reisner, 2016). The association between gender-affirming treatments and ED symptoms in TGD individuals is unclear, with few existing studies in this area. With the exception of one study, research indicates TGD adults receiving gender-affirming treatment report less ED symptoms compared to those waiting for interventions (Bandini et al., 2013; Jones et al., 2018; Murad et al., 2010). In TGD youth, findings are mixed. Avila and colleagues (2019) demonstrated no relationship between gender-affirming hormone use and ED symptoms while other studies demonstrate less severe body dissatisfaction and ED behaviors with this intervention (de Vries et al., 2014).

TGD youth may be at particular risk for EDs, due to unique experiences could promote long-established ED risk factors (e.g., body dissatisfaction), coupled with being in the age group with the greatest ED prevalence. Limited research regarding EDs in TGD youth prompted our study, with aims to: 1) describe the frequency and severity of ED symptoms in a sample of TGD youth seeking gender-affirming treatment; 2) compare ED symptoms in transgender females and transgender males; and 3) examine the association between gender-affirming hormone use and ED symptoms in this cohort. We hypothesized TGD youth would report ED behaviors and transgender females (youth assigned male at birth but identifying as female) would endorse higher levels of ED symptom severity compared to transgender males (youth assigned female at birth but identifying as males). We also hypothesized TGD youth receiving gender-affirming hormones would report less ED symptom severity than TGD youth not receiving hormones.

## Method

### Participants and procedure

Participants included 251 TGD youth (Mean age = 17.1 years, *SD* = 2.87, age range = 12–24) seeking gender-affirming treatment at a large pediatric medical center in the Midwestern United States between January 2015 and September 2018. Most participants in our sample (*n* = 181, 72.1%) identified as transgender male (identifying as male but assigned female at birth) and 70 (27.9%) identified as transgender female (identifying as female but assigned male at birth). Three youth identified as non-binary but were excluded from analyses given low sample size.

Retrospective chart review of electronic medical records was used to obtain patient characteristics, gender-affirming hormone use, and self-reported ED symptoms. This study was approved by the pediatric hospital’s institutional review board (IRB #2020-0768). Insofar as data used in our study was obtained via routine clinical care and inclusion of retrospective data posed no potential harm to patients, informed consent was waived by the IRB. All data was deidentified and stored separate from patient identifying information to maintain patient anonymity.

### Chart review

#### Clinical characteristics

The following data was extracted via retrospective chart review: gender identity (transgender female, transgender male, or gender non-binary), age, race, ethnicity, body mass index (BMI), and use of gender-affirming hormones prior to the initial clinic visit.

#### ED symptoms

TGD youth completed the Eating Disorder Examination-Questionnaire (EDE-Q; (Fairburn & Beglin, 1994)) at the initial transgender clinic visit. The EDE-Q is given as part of routine treatment in the institutional transgender clinic to assess for EDs and guide clinical care. The EDE-Q is a self-report questionnaire measuring ED attitudes and behaviors over the past 28-days (Fairburn & Beglin, 1994), is widely used (Lavender et al., 2010; Mond et al., 2006), and demonstrates adequate validity with cisgender individuals (Lavender et al., 2010; Mond et al., 2006). There is promising preliminary support for the use of the EDE-Q among TGD youth (Peterson et al., 2020) and adults (Nagata et al., 2020). The Global EDE-Q score (the average of the four EDE-Q subscales – Restraint, Weight Concern, Shape Concern, and Eating Concern) and individual EDE-Q items reflecting specific ED behaviors (i.e., objective and subjective binge eating episodes, self-induced vomiting, laxative use, and compensatory exercise (items 13–18)), were used in analyses. Further, the portion of youth reporting EDE-Q scores above the clinical cut-off of 4 (i.e., indicative of a potential ED; Mond et al., 2006) was assessed. Only the Global EDE-Q score was used given research suggesting EDE-Q is most valid when using a single factor structure (Global EDE-Q) (Allen et al., 2011; Aardoom et al., 2012; Peterson et al., 2020). Internal consistency for the Global EDE-Q was acceptable in our sample (α = .95).

### Statistical analyses

#### Participant characteristics

General descriptive analyses were conducted for participant characteristics, ED behaviors (EDE-Q items 13–18) and the Global EDE-Q (e.g., mean, standard deviation, and frequencies [for items 13-18, meeting ED cut-off].

##### ED symptoms among transgender females versus transgender males and associations with gender-affirming hormone use

A 2 × 2 ANCOVA controlling for age and BMI was conducted to examine differences between gender identity (transgender male, transgender female) and gender-affirming hormone use (yes/no) on Global EDE-Q scores. BMI and age were included as covariates in analyses given research support that these variables are associated with EDE-Q scores (Rø et al., 2012).

##### Frequency of ED behaviors among transgender females versus transgender males

To assess differences between transgender females and transgender males on frequency of ED behaviors zero-inflated negative binomial regression or negative binomial regression (when the zero inflated model was not significant) using Monte Carlo estimation (500 bootstrapping sample) were used since count data was not normally distributed (Long & Freese, 2006). Preliminary zero-inflated and negative binomial regressions were conducted and included gender identity (transgender female, transgender male), age, and BMI. Response variables were objective binge eating, subjective binge eating, self-induced vomiting, laxative use, and compensatory exercise. Age and BMI were not significant predictors, so analyses were rerun without including these variables.

##### Frequency of ED behaviors among transgender females and transgender males including the association of gender-affirming hormone use

Additional zero-inflated and negative binomial regressions were run including gender-affirming hormone use and gender identity as predictors of the same five ED behaviors. Lastly, Chi Square Analyses or Fisher’s Exact Tests (if cell sizes were less than five) were conducted to examine whether any engagement (zero versus any engagement) in ED behaviors was associated with gender-affirming hormone use and gender identity.

##### Statistical software

All analyses were conducted using SPSS version 25 (IBM Corp, 2017) except for negative binomial and zero-inflated negative binomial regressions where Mplus version 8.3 (Muthén & Muthén, 1998-2017) was used.

## Results

### Participant characteristics

Transgender females were significantly older than transgender males, *t*(249) = 2.54, *p* = .01. There were no significant differences in BMI between transgender females and transgender males, *t*(249) = −1.66, *p* = .10. Approximately 28% of TGD youth (*n* = 53) reported prior use of gender-affirming hormones, including estrogen (*n* = 14) and testosterone (*n* = 39). For 63 TGD youth, data regarding use of gender-affirming hormones was not available in the electronic medical record. See Table 1 for demographic details.

**Table 1.** Descriptives of demographics.

	Transgender males (*n* = 181)	Transgender females (*n* = 70)	Total (*n* = 251)
	*M* (*SD*)	*M* (*SD*)	*M* (*SD*)
Age	16.77 (2.74)	17.79 (3.12)	17.06 (2.87)
BMI	26.87 (7.88)	25.05 (6.49)	26.28 (7.55)
EDE-Q	1.34 (1.17)	1.55 (1.50)	1.39 (1.27)
	*n* (%)	*n* (%)	*n* (%)
Ethnicity			
Non-hispanic	130 (96.3)	43 (97.7)	173 (96.6)
Hispanic	5 (3.7)	1 (2.3)	6 (3.4)
Race			
White	122 (89.7)	40 (90.9)	162 (90.0)
Black	3 (2.2)	3 (6.8)	6 (3.3)
Asian	3 (2.2)	1 (2.3)	4 (2.3)
Other	8 (5.9)	0 (0.0)	9 (4.4)

### ED symptom severity comparison among transgender youth

While the mean EDE-Q Global score among TGD youth was 1.39 (*SD* **=** 1.27), 5.2% (*n* = 13) of our sample scored at or above the clinical cut-off score of 4.00 (Mond et al., 2008). Gender identity was not associated with EDE-Q cut-off scores, (*p* = .76, OR = 0.86, 95% CI [0.26, 2.90]). In evaluating specific ED behaviors in the past 28-days, 39 TGD youth (19.2%) reported at least one objective binge episode, 36 (17.7%) endorsed at least one subjective binge episode, 7 (3.4%) reported at least one episode of self-induced vomiting, 19 (8.8%) reported laxative use at least once, and 33 (16.3%) reported compensatory exercise at least once. See Table 2 for breakdown by gender identity.

**Table 2.** Descriptives of ED behaviors among transgender females and transgender males.

	Transgender females	Transgender males
**Subjective binge episodes**		
% Endorsed	23.2	15.6
Mean (SD)	2.82 (8.15)	1.14 (4.71)
Minimum – Maximum	0–38	0–28
**Objective binge episodes**		
% Endorsed	23.6	17.6
Mean (SD)	2.40 (6.32)	0.80 (3.21)
Minimum – Maximum	0–28	0–28
**Self-induced vomiting**		
% Endorsed	9.1	1.4
Mean (SD)	0.22 (0.76)	0.11 (0.96)
Minimum – Maximum	0–4	0–10
**Laxative use**		
% Endorsed	15.3	6.4
Mean (SD)	0.58 (3.81)	0.01 (0.08)
Minimum – Maximum	0–28	0–1
**Compensatory exercise**		
% Endorsed	12.9	16.2
Mean (SD)	0.91 (4.00)	1.23 (4.08)
Minimum – Maximum	0–28	0–35

% endorsed = percent of group reporting a frequency of 1 or greater of variable.

### ED severity and gender-affirming hormone use

After controlling for BMI, *F*(1, 165) = 3.19, *p* = .08 and age, *F*(1, 165) = 0.04, *p* = .84, there were no significant differences between transgender females and transgender males, *F*(1, 165) = 2.98, *p* = .09, Cohen’s *d* = 0.16, 95% CI of Cohen’s *d* [−0.18, 0.50] on Global EDE-Q score. There was also not a significant difference on Global EDE-Q related to gender-affirming hormone use, *F*(1, 165) = 3.35, *p* = .07, Cohen’s *d* = .30, 95% CI of Cohen’s *d* [−0.64, 0.04]. ED symptoms were not significantly different between TGD youth receiving gender-affirming hormones (*M* = 1.67, *SD* = 1.51) and those not receiving gender-affirming hormones (*M* = 1.29, *SD* = 1.15). The interaction between gender identity and gender-affirming hormone use was also non-significant, *F*(1, 165) = 2.42, *p* = .12. There was no difference in ED symptoms among transgender females and males when gender-affirming hormone use was included as a covariate (*p* > .05).

### ED behaviors among TGD youth

Zero-inflated and negative binomial regressions results indicated gender identity did not relate to different frequency in objective or subjective binge episodes, self-induced vomiting, laxative use, or compensatory exercise (all *p*s > .05). When gender-affirming hormone use was included as a predictor variable, neither gender-affirming hormone use or gender identity were significant predictors of ED behaviors (all *p*s > .05).

There was a significant interaction between gender identity and gender-affirming hormone use and objective binge episodes when assessing associations between frequency of any ED behavior and gender-affirming hormone use. A greater portion of transgender females using gender-affirming hormones (*n* = 7, 50.0%) reported objective binge episodes compared to transgender males using gender-affirming hormones (*n* = 4, 10.8%), Fisher’s Exact, *p* = .005, OR = 0.12, 95% CI [0.30, 0.53]. While there were no significant associations with gender-affirming hormone use and objective binge episodes among transgender males (all *ps* > .05), among transgender females, there was an association with gender-affirming hormone use and objective binge episodes, χ^2^(1) = 5.54, *p* = .03, OR = 4.83, 95% CI [1.23, 18.98]. Six (17.1%) transgender females who were not using gender-affirming hormones reported objective binge episodes while seven (50.0%) of transgender females using gender-affirming hormones reported objective binge episodes. There were no other significant interactions between gender-affirming hormone use and other ED behaviors (all *p*s > .05).

A 2 × 2 Chi Square test indicated there was no significant association with gender identity and any ED behaviors (all *p*s > .05) after collapsing gender-affirming hormone use. A Fisher’s Exact test indicated gender identity was associated with self-induced vomiting, *p* = .02, OR = 0.14, 95% CI [0.03, 0.73]. Five (9.1%) of transgender females endorsed at least one instance of self-induced vomiting compared to transgender males where only 2 (1.4%) reported any self-induced vomiting. In assessing associations with gender-affirming hormone use and ED behaviors, there were no significant associations overall with gender-affirming hormone use on any ED behaviors (all *p*s > .05) when gender was collapsed. See Table 3 for proportion of transgender females and transgender males as well as TGD youth using gender-affirming hormones or not using gender-affirming hormones on all ED behaviors.

**Table 3.** Portion of TGD youth reporting ED behaviors based on gender identity and gender-affirming hormone use.

	ED behavior	Gender-affirming hormones		No gender-affirming hormones		Total endorsed‡	Total not endorsed‡
		Endorsed	Not endorsed	Endorsed	Not endorsed		
Subjective binge episode							
	Transgender female	4 (28.6%)	10 (71.4%)	8 (22.2%)	28 (77.8%)	12 (24.0%)	38 (76.0%)
	Transgender male	5 (13.5%)	32 (86.5%)	16 (18.0%)	73 (82.0%)	2 (16.7%)	105 (83.3%)
	Total hormones^†^	9 (7.6%)	42 (82.4%)	24 (19.2%)	101 (80.8%)		
Objective binge episode							
	Transgender female	7 (50.0%)	7 (50.0%)	6 (17.1%)	29 (82.9%)	13 (26.5%)	36 (73.5%)
	Transgender male	4 (10.8%)	33 (89.2%)	19 (21.1%)	71 (78.9%)	23 (18.1%)	104 (8.19%)
	Total hormones	11 (21.6%)	40 (78.4%)	25 (20.0%)	100 (80.0%)		
Self-induced vomiting							
	Transgender female	2 (14.3%)	12 (85.7%)	3 (8.5%)	32 (91.4%)	5 (10.2%)	44 (89.8%)
	Transgender male	1 (2.7%)	36 (97.3%)	1 (1.1%)	89 (98.8%)	2 (1.6%)	125 (98.4%)
	Total hormones	3 (5.9%)	48 (94.1%)	4 (3.2%)	121 (96.8%)		
Laxative use							
	Transgender female	3 (21.4%)	11 (78.6%)	5 (12.8%)	34 (87.2%)	8 (15.1%)	45 (84.9%)
	Transgender male	2 (5.1%)	37 (94.9%)	6 (6.3%)	90 (93.8%)	8 (5.9%)	127 (94.1%)
	Total hormones	5 (9.4%)	48 (90.6%)	11 (8.1%)	124 (91.9%)		
Compensatory exercise							
	Transgender female	4 (29.6%)	10 (71.4%)	4 (11.4%)	31 (88.6%)	8 (16.3%)	41 (83.7%)
	Transgender male	7 (18.9%)	30 (81.1%)	16 (17.8%)	74 (82.2%)	23 (18.1%)	104 (81.9%)
	Total hormones	11 (21.6%)	40 (78.4%)	20 (16.0%)	105 (84.0%)		

†Collapsing gender, percentage of individuals engaging in any or no ED behaviors based on gender-affirming hormone use, ‡Collapsing gender-affirming hormone use, percentage of individuals engaging in any or no ED behaviors based on identifying as Transgender female and Transgender male.

## Discussion

This study aimed to describe and compare ED symptoms among transgender females and transgender males, and to examine the association between gender-affirming hormone use and ED symptom severity in TGD youth seeking gender-affirming care. Approximately 5% of TGD youth in our sample endorsed ED symptoms at or above the clinical cut-off on the EDE-Q (Mond et al., 2006), consistent with prior studies among TGD youth and adults (Feder et al., 2017; Ferrucci et al., 2022), and higher than those evident in community samples of cisgender youth (e.g., Penelo et al., 2013).

Of particular concern, one-fifth of our sample endorsed at least one ED behavior in the last 28-days, including subjective binge eating (17.7%), objective binge eating (19.2%), self-induced vomiting (3.4%), laxative use (8.8%), or compensatory exercise (16.3%). Our study did not include a community sample of cisgender youth for comparison, but these rates appear significantly higher than those cited previously (Mond et al., 2014; Penelo et al., 2013. For example, laxative use in our sample was reported in 15.3% and 6.4% of transgender females and transgender males, respectively, compared to published rates of 2.1% in adolescent and young adult (presumably) cisgender females and 0.2% in (presumably) cisgender males (Mond et al., 2014). Subjective binge eating was reported by 23.2% of transgender females and 15.6% of transgender males in our study versus 14.5% of females and 4.2% of males in presumed cisgender community samples (Mond et al., 2014). Heightened ED symptoms among TGD youth may relate to established ED risk factors among adolescents generally (e.g. thin- and muscle-ideal internalization) with the added influence of risk factors specific to identifying as TGD including gender dysphoria and minority stress (Cusack et al., 2021; Hembree et al., 2017; McClain & Peebles, 2016; van de Grift et al., 2016).

When comparing, transgender females and males, self-induced vomiting and objective binge eating were more prevalent in transgender females versus transgender males (9.1% versus 1.4% and 23.6% versus 17.6%, respectively), as has been reported in TGD adults (Darcy et al., 2012; Mantilla & Birgegård, 2016; Watson et al., 2017). Inconsistent with prior studies suggesting higher Global EDE-Q scores in adult samples of transgender females versus transgender males (e.g., Testa et al., 2017; Vocks et al., 2009), we found no differences in Global EDE-Q scores suggesting similar levels of ED symptoms severity among TGD youth in our sample. Although speculative, this could reflect different developmental stages and experiences in TGD youth versus adults. For youth, puberty is at the forefront and unless blocked by hormones, universal for transgender females and males. Characterized by the development of secondary sex characteristics, TGD youth must navigate the pubertal body changes may be incongruent with their identified gender, surely intensifying body dissatisfaction, regardless of being transgender female or male. TGD adults, having surpassed puberty, may experience challenges unique to being transgender female or transgender male. For example, transgender female adults have indicated greater difficulties “passing” as their identified gender than transgender males (Vocks et al., 2009), plausibly prompting greater ED symptom severity in efforts to appear feminine.

Approximately 25% of TGD youth in our study reported a history of gender-affirming hormone use. Among transgender females, 50% of those taking gender-affirming hormones endorsed at least one objective binge eating episode in the last 28-days compared to only 17.1% of transgender females not taking hormones. This could reflect the relation between estrogen (via gender-affirming hormones) and increased leptin levels (Resmini et al., 2008), as leptin has been associated with binge eating (Miller et al., 2014) and appetite regulation (McDuffie et al., 2004), and warrants further examination.

Inconsistent with our hypothesis and prior findings in TGD adults (de Vries et al., 2011; Fisher et al., 2014; Jones et al., 2018; Testa et al., 2017), gender-affirming hormone use was not associated with overall ED symptomatology in our sample. This could be due to our examination of hormone use only, while other studies have examined diverse medical treatments, including hormone replacement, chest and genital surgeries, hair removal, and hysterectomies (de Vries et al., 2011; Fisher et al., 2014; Jones et al., 2018; Testa et al., 2017). It is also conceivable hormone use in TGD youth is less impactful on ED symptoms than in TGD adults or in comparison to using other gender-affirming treatments. It is possible the amount of time or stage of gender-affirming treatment is associated with ED behaviors and severity. Our retrospective study design did not facilitate standardized assessment of the timeline and duration of hormone use, including relation to puberty onset. It can take months before associated physical changes are visible (Hembree et al., 2017), and a relationship between gender-affirming hormones and ED symptoms could be moderated by *duration* of hormone use or suppression of pubertal development incongruent with one’s gender identity. Thus, starting gender-affirming hormones alone may not be associated with lower ED symptoms. In addition to timing of gender-affirming care, cognitive appraisal may impact improvements and/or protection against ED symptoms (e.g. a sense of gender affirmation from others, the belief one appears as their identified gender, or feeling as though one has completed their gender transition). Finally, a relatively low number of youth in our sample had used gender-affirming hormones. This may have limited statistical power to replicate prior findings in adults.

### Limitations and future directions

Our study demonstrates TGD youth endorse significant ED behaviors and that transgender females endorsed greater self-induced vomiting and objective binge eating episodes compared to transgender males. Our study failed to demonstrate differences in ED symptoms related to gender-affirming hormone use and was one of the first studies to assess this. Yet, our exploratory design was limited in scope; data was acquired via chart review prohibiting standardized assessment of gender dysphoria in our sample. We can only speculate most TGD youth who seek gender-affirming treatment at a specialized transgender clinic experience distress associated with gender incongruence. This study only looked at one aspect of gender-affirmative care, gender-affirming hormones, and evaluated if patients had started gender-affirming hormone replacement at the time of their initial transgender clinic visit. It was unclear whether youth in our sample planned to initiate other forms of gender-affirming care in the future. Future studies should more comprehensively examine the association between all forms of gender-affirming treatment and ED longitudinally, particularly in the context of puberty.

We also did not assess for psychiatric diagnoses or psychotropic medication use, given inconsistent documentation of these variables in patient charts. Thus, our study design prohibited a comprehensive understanding of overall psychological functioning of our sample. Additionally, the sample was not inclusive of all gender identities (only three youth identified as gender nonbinary and dropped due to statistical needs) nor did it include many gender expansive youth of color. These limitations prohibit us from drawing conclusions about ED symptoms beyond a somewhat homogenous sample of TGD youth.

Whereas preliminary studies suggest the EDE-Q to be valid and reliable among TGD individuals (Nagata et al., 2020; Peterson et al., 2020), EDE-Q items may not adequately represent unique symptoms experienced among TGD individuals. For example, Witcomb and colleagues (2015) found TGD individuals present with different body image concerns and motivators for engagement in ED behaviors compared to cisgender peers (Witcomb et al., 2015). The EDE-Q may miss nuanced features of body dissatisfaction experienced by TGD individuals, such as drive for muscularity (Rabito Alcón & Rodríguez Molina, 2016) and gender dysphoria. Further, gender identity is not a binary construct; thus body dissatisfaction may present differently among individuals who identify as gender non-binary or gender fluid compared to dichotomized transgender female and transgender male counterparts.

### Conclusions and future directions

Approximately one in four treatment-seeking TGD youth in our sample reported recent engagement in ED symptoms, with 5% endorsing severe symptoms, as indicated by scoring at or above the recommended clinical cut-off on the EDE-Q (Mond et al., 2008), and 20% of our sample endorsed engaging in at least one ED behavior in the past month. These findings highlight the importance of ED assessment and treatment among TGD youth seeking care, particularly because TGD youth may already be at greater risk for ED development given their age and pubertal development. We found minimal differences in ED symptoms in transgender females compared to transgender males and notably, gender-affirming hormone use was unrelated to overall ED symptoms in our sample.

Despite limitations, our study is novel as it included a large clinical sample of TGD youth seeking various ranges of gender-affirming treatment at a pediatric medical center. Many previous studies evaluating the relationship between gender-affirming hormone use and ED symptoms assessed individuals who had either undergone medical intervention or intended to begin treatment. Thus, we used a more representative sample of TGD youth as some TGD individuals will not elect to undergo any medical intervention as they seek gender-affirming treatment and transition (McClain & Peebles, 2016).

Future research should delineate the association between gender dysphoria, gender-affirmation treatment use, and ED symptoms among TGD youth, including longitudinal changes in ED symptoms in relation to gender dysphoria, puberty, and types and stages of gender-affirmation treatment, and the mechanisms by which gender dysphoria and gender-affirmation treatments impact ED risk. These studies could further guide the evidence-based nature of clinical and medical interventions for TGD youth and could garner even further support for existing WPATH Standards of Care (Coleman et al., 2022). Finally, gender identity is a spectrum, and a more comprehensive representation of all gender identities, beyond transgender females and transgender males, and ED risk is crucial. Individuals identifying as non-binary may report differences in gender dysphoria and body image concerns than transgender individuals identifying on the binary (Galupo & Pulice-Farro, 2020; Johnson et al., 2020) and gender non-binary individuals represent 35% of all individuals identifying as transgender (Matsuno & Budge, 2017).
